# Applying machine-learning to rapidly analyze large qualitative text datasets to inform the COVID-19 pandemic response: comparing human and machine-assisted topic analysis techniques

**DOI:** 10.3389/fpubh.2023.1268223

**Published:** 2023-10-31

**Authors:** Lauren Towler, Paulina Bondaronek, Trisevgeni Papakonstantinou, Richard Amlôt, Tim Chadborn, Ben Ainsworth, Lucy Yardley

**Affiliations:** ^1^School of Psychology, University of Southampton, Southampton, United Kingdom; ^2^School of Psychological Science, University of Bristol, Bristol, United Kingdom; ^3^Department of Health and Social Care, Office for Health Improvement and Disparities, London, United Kingdom; ^4^Institute for Health Informatics, University College London, London, United Kingdom; ^5^Department of Experimental Psychology, Division of Psychology and Language Sciences, University College London, London, United Kingdom; ^6^Behavioural Science and Insights Unit, UK Health Security Agency, London, United Kingdom; ^7^Department of Psychology, University of Bath, Bath, United Kingdom; ^8^National Institute for Health Research Biomedical Research Centre, Faculty of Medicine, University of Southampton, Southampton, United Kingdom

**Keywords:** public health, interventions, qualitative analysis, machine learning techniques, triangulation

## Abstract

**Introduction:**

Machine-assisted topic analysis (MATA) uses artificial intelligence methods to help qualitative researchers analyze large datasets. This is useful for researchers to rapidly update healthcare interventions during changing healthcare contexts, such as a pandemic. We examined the potential to support healthcare interventions by comparing MATA with “human-only” thematic analysis techniques on the same dataset (1,472 user responses from a COVID-19 behavioral intervention).

**Methods:**

In MATA, an unsupervised topic-modeling approach identified latent topics in the text, from which researchers identified broad themes. In human-only codebook analysis, researchers developed an initial codebook based on previous research that was applied to the dataset by the team, who met regularly to discuss and refine the codes. Formal triangulation using a “convergence coding matrix” compared findings between methods, categorizing them as “agreement”, “complementary”, “dissonant”, or “silent”.

**Results:**

Human analysis took much longer than MATA (147.5 vs. 40 h). Both methods identified key themes about what users found helpful and unhelpful. Formal triangulation showed both sets of findings were highly similar. The formal triangulation showed high similarity between the findings. All MATA codes were classified as in agreement or complementary to the human themes. When findings differed slightly, this was due to human researcher interpretations or nuance from human-only analysis.

**Discussion:**

Results produced by MATA were similar to human-only thematic analysis, with substantial time savings. For simple analyses that do not require an in-depth or subtle understanding of the data, MATA is a useful tool that can support qualitative researchers to interpret and analyze large datasets quickly. This approach can support intervention development and implementation, such as enabling rapid optimization during public health emergencies.

## 1. Introduction

Qualitative research plays a vital role in public health, intervention development and implementation research by enabling researchers to develop an informed understanding of the attitudes, perceptions and contextual factors relevant to planning and delivering effective and acceptable health interventions (1, 2). However, most qualitative approaches (such as interviews, focus groups and observation studies) are resource intensive and time-consuming, requiring months or years to collect and analyze rich, in-depth data. Consequently, most qualitative approaches have traditionally been based on studies of relatively small, purposively selected samples (3). Whilst this kind of in-depth approach has enormous benefits in terms of generating nuanced insights for the purpose of theory-building, it is less suitable for some potential applications of qualitative methods. In particular, less resource intensive methods are needed in order to analyze the wealth of qualitative data that can be generated by automated online data collection (for example, of free text responses to population surveys). Whilst computational and automated approaches are commonly used in the field of epidemic modeling to monitor disease spread and adoption of preventative behaviors by members of the public [e.g., (4)], these methods do not provide sufficient insight into perceptions of preventative behaviors and individual decision-making processes that qualitative approaches offer.

Recent advances in technology have facilitated the automatic processing of text-based qualitative datasets, via natural language processing (NLP), a subfield of artificial intelligence. NLP algorithms can quickly produce “triaged” natural text outputs, that have the potential to substantially reduce the amount of text to be examined by research teams whilst remaining meaningful (5). NLP has been applied in several areas of healthcare research: extracting information from electronic healthcare records (6, 7), coding interview transcripts about male health needs (8), or early detection of depression in social networks (9). A direct comparison of an NLP approach which used lexicon-based clustering in WordNet with human-only qualitative analysis analyzed answers from 84 participants to short open-ended text message survey questions (10). They found that NLP generated similar findings although was not of as high quality, and could be used to in combination with human qualitative analysis to provide more detail.

Indeed, the importance of the input of experienced qualitative researchers to NLP-assisted qualitative data analysis must not be overlooked. Findings by Guetterman et al. (10) highlight how experienced qualitative researchers bring knowledge of contextual, theoretical, and sociocultural factors that cannot be replicated by NLP-only approaches. Whilst previous studies show how NLP methods can be used to support deductive approaches where an *a priori* coding framework is in place (11), there is often a need to conduct “bottom-up” inductive and exploratory analyses where ideas are formed from the data itself, particularly when developing new public health interventions or adapting existing interventions to new situations or populations. Inductive qualitative analysis allows researchers to explore relevant issues and topics as guided by members of the relevant population, and generate new ideas in a data-driven way (12, 13). In this project, we therefore aimed to explore the use of a different specific NLP approach which integrates human and exploratory NLP analysis– which we have termed “Machine-Assisted Topic Analysis” (MATA) – to allow expert qualitative researchers to look at large, real-world datasets in a timely manner.

MATA assists qualitative researchers by summarizing major patterns in the text according to generative models of word counts – known as topic models (14). Topic models are able to automatically infer latent topics from text. This means the model assumes that the documents consist of a combination of underlying topics and can be represented as such. Topic models allow for machine-assisted reading of text datasets through creating and extracting the main themes that underlie a corpus and mapping them onto the individual documents. They are particularly useful as tools to analyze large volumes of free-text responses to questions in a data-driven way, in order to summarize the main families of responses. The approach used in this study is based on an application of the Structural Topic Model (14, 15) in particular. The STM is a general framework for topic modeling that is differentiated from other topic modeling methodologies by its ability to enable researchers to include additional variables at the document level, such as the date a document was created or the demographics of the person who created it, as covariates in a topic model. This way the relationships of these variables to specific topics can be estimated and examined or used to run subgroup analyses. Those variables are further used to explain variance in topic prevalence, so affect the frequency with which a topic is discussed. As a result, their inclusion improves inference and qualitative interpretability and also affects the topical content (14). Structural topic models are able to identify patterns, and qualitative researchers can then use the output to extract meaning, interpret and summarize the topics.

Within the context of COVID-19, several NLP researchers have identified NLP as a potentially effective tool for rapid analysis of large-scale text-based datasets in order to meet the rapidly shifting public health needs during a pandemic (11, 16, 17). For example, NLP approaches could allow the rapid analysis of views and experiences of public health interventions (such as infection tracking tools, or public health messaging services) via survey response, allowing teams to improve interventions in real-time as issues arise – which can be vital given the rapidly changing context of a worldwide pandemic (3, 18). However, previous comparisons between exploratory NLP methods and human-only qualitative analyses have mostly been conducted on relatively small sample sizes (8, 10). Therefore, there is a need to assess how NLP methods can inductively analyze large datasets for studies with exploratory aims. One such study using a large dataset demonstrated that supervised machine learning approaches could effectively complement human hand-coding (19). The current study builds on Nelson’s work by attempting to demonstrate how NLP methods can be applied to ‘real world’ participant data in a rapid-response situation, providing further evidence for the validity and utility of the method.

Germ Defence is a digital behavior change intervention that aims to improve infection control behaviors during the COVID-19 pandemic (20). In order to remain as effective as possible, Germ Defence was iteratively updated throughout the pandemic, as health guidelines and contextual factors (e.g., virus prevalence, vaccine uptake) changed (18). During the intervention, some website users provided feedback about the content and design, and we used this data to perform separate qualitative analyses using MATA and human-only analysis. We aimed to explore similarities and differences between findings of the two methods, and to compare the person-hours required to conduct each form of analysis, in order to assess the potential value and trustworthiness of MATA for large-scale public health intervention evaluation and optimization.

## 2. Methods

### 2.1. Participants

Inclusion criteria were users of the Germ Defence website who were over the age of 18 and able to give informed consent. Between 18th November 2020 until 3rd January 2021, a total of 2,175 people consented to the survey, 1,472 of which responded to at least one open-ended question. During this time, a second national lockdown was in place in the UK, which was replaced by the reintroduction of the tiered system on 2nd December 2020. Data collection ended prior to the third national lockdown on 6th January 2021. Table 1 shows the demographic characteristics of the sample.

**Table 1:** Demographic characteristics of the sample (*N* = 1,472).

Demographics	*N*	%
*Who do you live with*		
Alone	304	20.7
With children under 16	176	12.0
With family all over 16	889	60.4
With people not related to me	73	5.0
Blank	30	2.0
*Increased risk of severe illness (self or household member)*		
Yes	861	58.5
No	535	36.3
Blank	76	5.2
*Possibility of current COVID-19 infection (self or household member)*		
Yes	69	4.7
No	1,335	90.7
Blank	68	4.6
*Age*		
18–25	10	0.7
26–40	76	5.2
41–60	524	35.6
61–70	471	32.0
70+	324	22.0
Blank	67	4.5
*Sex*		
Female	972	66.0
Male	423	28.7
Other or prefer to self-describe	4	0.3
Prefer not to say	3	0.2
Blank	70	4.8
*Ethnicity*		
White	1,331	90.4
Black African	2	0.1
Black Caribbean	5	0.3
Black (other)	2	0.1
Indian	9	0.6
Pakistani	4	0.3
Bangladeshi	1	0.1
Chinese/Southeast Asian	6	0.4
Asian (other)	6	0.4
Other	28	1.9
Prefer not to say	8	0.5
Blank	70	4.8
*Education*		
Before finishing school	33	2.2
After finishing school	643	43.7
After finishing university	353	24.0
After postgraduate studies	280	19.0
Blank	163	11.1

Participants who selected “Other” categories for ethnicity were able to give an additional open-text response. Most who selected this category were from mixed backgrounds, but some specified themselves as, for example, White Armenian, Turkish/Cypriot, or Nepalese etc.

### 2.2. Measures

To gather demographic data, closed questions were asked pertaining to age, sex, ethnicity, education, household size, whether the user or someone else in the household is at increased risk of severe illness if they caught COVID, and whether there could be a current COVID case within the household (experiencing symptoms or contact with confirmed case). Feedback was collected as free-text responses to two questions: “What was helpful about the information on the Germ Defence website?” and “What did you not find helpful about the information on the Germ Defence website?” Responses to these questions provide a rich dataset of recommendations that can be used to improve the website and guidance provided. The goal of this data collection and analysis was to investigate perceptions of the Germ Defence website, with particular attention to the acceptability of the intervention and the advice it provides.

### 2.3. Procedure

After they had completed at least one of the two main sections of the intervention (handwashing or reducing illness), visitors to the Germ Defence website received a pop-up asking if they might be interested in taking a survey to help improve the website. The invitation was presented as seeking information on users’ views on protecting themselves from Coronavirus, and their thoughts on the Germ Defence website. Users could then follow a link to the study information sheet, consent form, and the online questionnaire hosted on Qualtrics. Ethical approval was granted by the University of Southampton Psychology Ethics Committee (ID: 56445).

### 2.4. Data analysis

We analyzed the data in two ways; human-only qualitative analysis and MATA.

#### 2.4.1. Human-only qualitative analysis

The human-only analysis was conducted using a codebook thematic analysis (TA) approach using template analysis techniques (21–23) whereby the coding template was applied to the data deductively by several coders, and the unit of analysis was free-text participant response. The coding team was made up of an experienced qualitative researcher and lecturer at the University of Southampton (LT), and a group of 6 voluntary research assistants (VRAs) made up of both undergraduate and postgraduate Psychology students at the Universities of Southampton and Bath.

The initial codebook had been developed through the researchers’ (LT) contextual knowledge, involvement in collating feedback for the person-based approach (PBA) development of the Germ Defence intervention, and derived from smaller-scale survey data and formal TA of qualitative interviews with website users (24). Inductive coding was also implemented by the coders where relevant data did not currently fit with existing codes. Any proposed additional inductive codes identified during coding were discussed with the group as soon as possible, so that each coder could keep it in mind for their own coding. However, as the process went on, many of these inductive codes were deemed too thin to remain as standalone codes (for example, “environmental concerns over waste, e.g., disposable masks” or “it’s too cold to ventilate”), and so they were merged together or with existing codes. As a result, some of the deductive codes evolved into broader, higher-level codes throughout the process than their original form. This process was done by discussion and interpretation of the code meaning by the team. Any disagreements between the team members throughout this process was resolved by discussion until agreement was reached.

LT then interpreted the shared meaning of the codes within the final framework and created the themes, which were discussed with the team. See Table 2 for further information on how the codebook was developed, and the procedures used in the human analysis. In the MATA, template analysis techniques were also used to analyze the topics generated by the STM, with each topic being the unit of analysis.

**Table 2:** Human-only analysis procedure and person-hours.

	Procedure	Hours (total person-hours)
Preparation	Each of the 7 coders were assigned ~210 participants, whose responses were transferred to the NVivo software package. LT set up the initial coding template based on a codebook developed and validated during previous analyses of Germ Defence data (24), previous survey data gathered from website users, and some initial data familiarization. Six VRAs were trained by LT in qualitative coding and using NVivo. This involved giving the VRAs an overview of the qualitative process and its aims, the coding process and the meaning of inductive and deductive coding, and previous qualitative analyses from the Germ Defence project.	25
Coding	Analyzed using a codebook TA approach, template analysis (23). The data were coded deductively onto the thematic codebook, though some inductive codes were integrated into the codebook upon discussion with the team.	95 (13.6 h per coder)
Validity checks	The first 50 survey respondents allocated to each trainee coder (23.81% of average total respondents per coder) were cross-checked, and any discrepancies were discussed in subgroups until agreement was reached, under supervision of LT.	14
Interpretation	LT interpreted the findings and created themes from the coding and discussed with the team. LT presented the results to the wider team, and made any adjustments based on discussion with the coders and wider team.	13.5
Total person hours		147.5

#### 2.4.2. Machine-assisted topic analysis

Structured data, such as date, age, sex, education level and ethnicity, were also collected and included in the models as covariates.

##### 2.4.2.1. Preparation

We pre-processed the data using R (version 3.5.2), and cleaned the free text responses using base R functions, the quanteda [version 2.0.1; (25)] and STM [version 1.3.3; (14)] packages. We deleted observations with missing values and duplicate data. The free-text responses were converted into token units using the quanteda package, after punctuation, symbols and numbers were removed. In this instance the tokens were individual words. Data pre-processing was completed by deleting stop words and stemming the tokens. Stemming is the process of reducing words to their root. This acts as a normalization of text data and helps reduce the size of the dictionary which speeds up processing.

##### 2.4.2.2. Coding and validity checks

As a topic modeling method, we implemented the Structural Topic Model (15). Prior to running the models we ran diagnostics to identify the optimal number of topics, according to both the relevant metrics and the aims of the analysis, focusing on the trade-off between semantic coherence and exclusivity [see (15) for a discussion on this method of evaluation]. We tested models with 5–40 topics and differing covariates in terms of semantic coherence score (see (26)), residuals and interpretability by human coders (see Supplementary material 1), separately for each question. Upon visually examining the plots in Supplementary material 2, we identified a Structural Topic Model with 25 topics to be optimal for addressing question A, “What was helpful about the information on the Germ Defence website?” whereas 15 topics were deemed to be optimal for addressing question B, “What did you not find helpful about the information on the Germ Defence website?” In both cases date, age, gender, ethnicity, and level of education were included as covariates. The model automated the equivalent of the coding stage of the analysis by assigning a number of labels to each document, by way of mapping them to topics. The code used for data preparation and modeling is publicly available in the figshare repository: https://figshare.com/articles/dataset/Germ_Defence_-_Machine_Assisted_Topic_Analysis/19514305.

##### 2.4.2.3 Interpretation: qualitative analysis of machine-generated data by trained, supervised coders

The outputs examined consisted of two main elements; the 10 most representative quotes for each topic and two lists of weighted words that constitute the topic. Different types of word weightings were generated with each topic where the following two types were analyzed in subsequent qualitative analysis: (1) Highest Prob (words within each topic with the highest probability) and (2) FREX (words that are both frequent and exclusive, identifying words that distinguish topics). Examples of outputs generated are presented in Supplementary material 3.

In order to analyze the model’s output systematically we analyzed it in two stages. In Stage 1, two researchers interpreted the output and agreed upon narrative labels for the topics (henceforth, MATA codes). In the first part of Stage 1 the analysis was blinded. In the second part the two researchers resolved any disagreements in the interpretation through discussion and agreed in the final topic labels. In Stage 2, the researchers analyzed the topics generated by the text analysis and created broader themes. Table 3 provides a breakdown of the steps of the MATA process, along with the person-hours that were spent on each step.

**Table 3:** Machine-assisted topic analysis approach and person-hours.

	Procedure	Hours
Preparation	Data cleaning and conversion of data to STM format	8
Coding	The structural topic model is run. The model infers the topics from the corpus of text and maps them back to individual response extracts, which are now automatically grouped into their assigned topics and represented as a distribution of them.	0
Validity checks	Diagnostic analysis and evaluation is conducted of models with 5–40 topics	4
Interpretation	Two coders interpret and apply narrative labels (codes) to the topics (stage 1). This procedure is conducted independently and blindly by the two coders to ensure accuracy and validity. The coders then create broader themes in collaboration through the process of thematic analysis to generate the final template (stage 2)	28 (9 h per coder)
Total person hours		40

#### 2.4.3. Triangulation

We conducted a formal triangulation in order to compare the results from both approaches. Specifically, we performed a methodological and investigator triangulation, as the results from two different analytical approaches performed by two different analysts were compared (27). Two research teams independently analyzed the Germ Defence data using the two methods described in the previous sections (MATA and human-only TA). A “convergence coding matrix” (28, 29) was created, and two researchers from these separate teams (LT and PB) independently triangulated the findings from both analyses. The codes were then compared with each other and categorized as either; agreement, complementarity, dissonance, or silence (28, 29). Agreement represented conceptual convergence between the analyses, and complementarity referred to a shared meaning or essence between the findings, but some unique nuances were present. Dissonance represented disagreement between the coding, and silence referred to a finding which was present in only one of the analyses. As such, codes were not considered dissonant with each other when they only represented difference of opinion within the sample, and not between the coding from the two methodologies. For example, the code ‘clear and simple’ from the human analysis was not considered dissonant with ‘wordy and repetitive’ from the MATA because alternative codes were present which agreed, such as ‘information was clear, concise, and easy to understand.’ The two analysts then compared and discussed their decisions and reached consensus on the findings.

## 3. Results

### 3.1. Person hours

The human qualitative analysis required significantly higher person hours to complete than the MATA (147.5 vs. 40). The only stage which less time in the human analysis than the MATA was the final interpretation stage, likely due to the familiarity with the data gained by coding the data “by hand” and the pre-existing coding template. In the MATA approach, the inference of the topics and the classification component of the analysis was conducted by the machine learning model. In this case, the final interpretation phase consisted of the two stages of generating narrative descriptions of the produced topics and following the process of thematic analysis. This was the first time the human coders came into contact with the data and thus this step was the most time-consuming one in the MATA.

### 3.2. Primary data analysis

The MATA results were centered on what users found helpful and unhelpful about the Germ Defence website. The themes representing what users found helpful were: 1. *Clear and easy to understand*, 2. P*rovision of new information and reminders*, 3. *Confirming and Reinforcing*. The themes representing what users found unhelpful were: 1. *Repetitive, simplistic, wordy, patronizing*, 2. *Lack of tailoring*, 3. Var*ious issues relating to usability, content and specific features*. For the human analysis, we found 3 main themes: (1) *layout and language style*, (2) *confidence in how to perform the behaviors*, and (3) *reducing all or nothing thinking*. These themes, and how they relate to each other, are presented in section 3.3 Triangulation. As the current study is concerned with the results of the triangulation between the two methods, further information on the results of the separate primary analyses can be found in Supplementary materials 4, 5.

#### 3.2.1. Machine-assisted topic analysis process: inclusion of topics

##### 3.2.1.1. What was helpful about the information on the germ defence website?

Of 25 topics analyzed qualitatively, 22 topics were included in the analysis as they provided substantial insights as expressed by the users’ feedback^-^. See Supplementary material 5 for a ranking of the machine-generated topics in terms of prevalence in the corpus for question A.

##### 3.2.1.2. What did you not find helpful about the information on the germ defence website?

Of 15 topics analyzed qualitatively, 13 topics were included in the analysis as they provided substantial insights as expressed by the users’ feedback^2^. See Supplementary material 5 for a ranking of the machine-generated topics in terms of prevalence in the corpus for question B.

**Figure 1:**
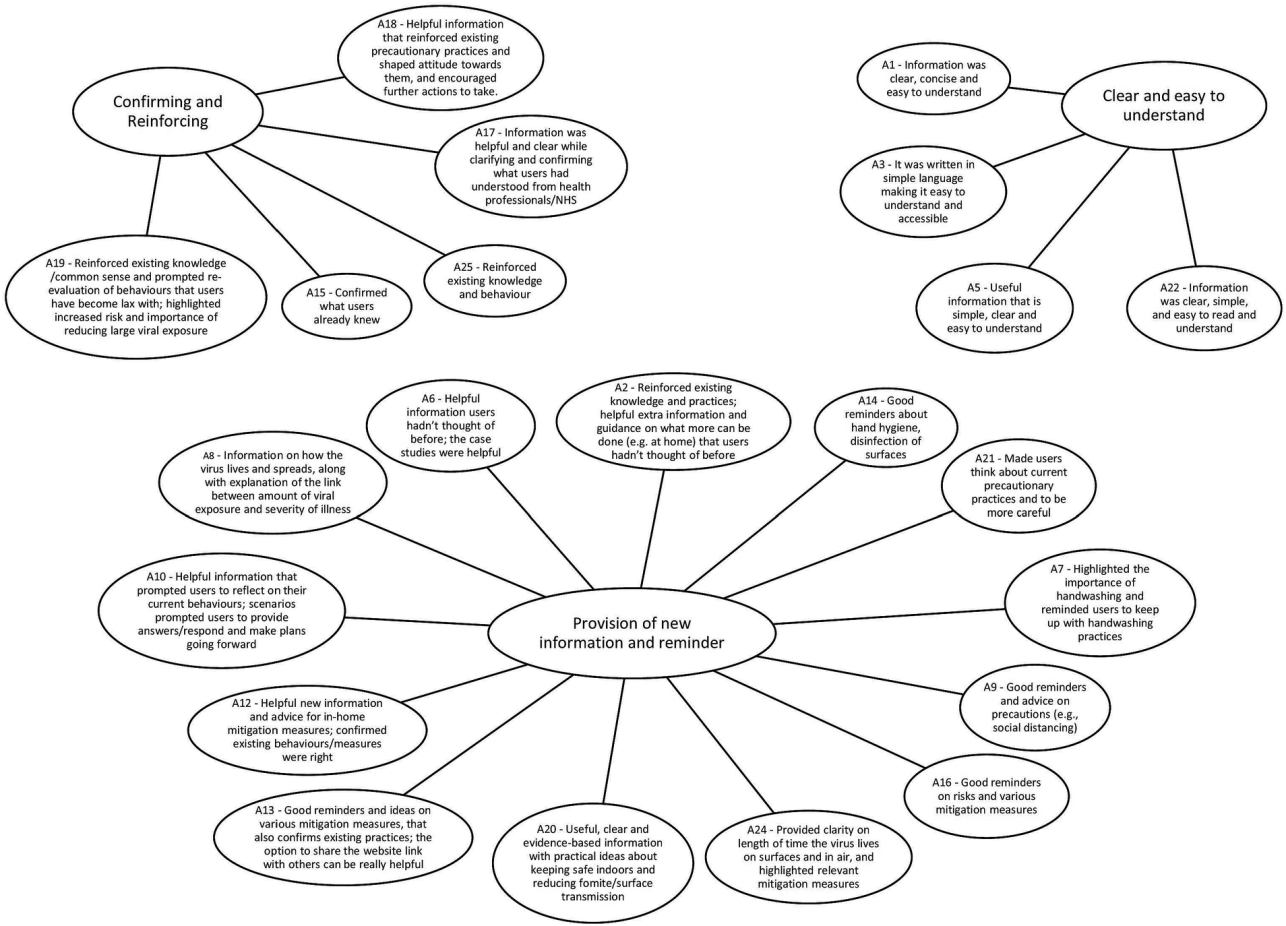
What was helpful about the information on the Germ Defence website? Summary of the topics (generated by the model, described by human) and the major themes (generated by human).

**Figure 2:**
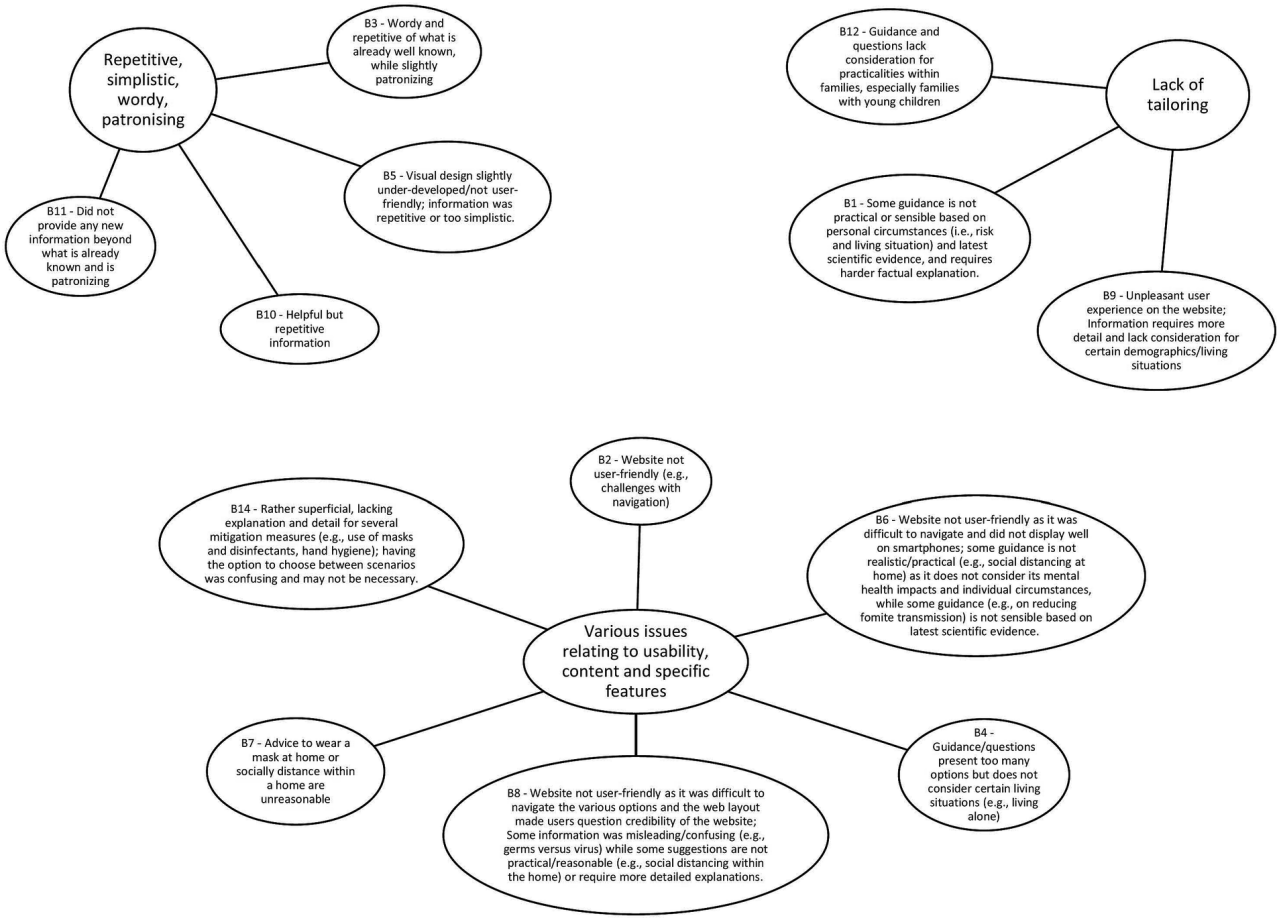
What did you not find helpful about the information on the Germ Defence website? Summary of the topics (generated by the model, described by human) and the major themes (generated by human).

The MATA codes from both corpora were grouped into major themes representing what users found helpful/unhelpful with the Germ Defence intervention (Figures 1, 2).

### 3.3. Triangulation

The codes generated from each form of analysis were categorized as either in agreement, or complementary to each other. We found no instances of dissonance or silence within the coding from the two methods. Table 4 presents the full results of the triangulation.

**Table 4:** Results of the triangulation between the human-only analysis and the MATA.

Human-only themes	Human-only codes	Triangulation with MATA codes	
		Agreement	Complementary
Layout and language style	Clear and simple	A1, A3, A5, A22	
	Not enough information	B9, B11, B14	
	Not streamlined or sophisticated	B5, B2, B6, B8	
	Too repetitive	B3, B5, B10	
	Too simplistic/patronizing	B3, B5, B11, B14	
Confidence in how to perform the behaviors	Clear practical advice and troubleshooting is helpful	A2, A6, A9, A10, A12, A13, A20, A24, A7, A21, A14, A18	B12, B9
	Feeling informed and reinforced by reliable sources is empowering	A12, A13, A16, A20, A7, A14, A25, A19, A17	A15
	Inconsistencies undermine confidence		A20, A17
Reducing all or nothing thinking	Trying to perform all the behaviors is exhausting		B12, B6
	Understanding that small changes matter is motivating		A8, A21, A19
	We should act according to risk	B1	A16, A19
	Some behaviors are very challenging in certain situations	B12, B1, B4, B6, B8	B9

#### 3.3.1. Instances of agreement

There was a high level of agreement between the findings of the human and MATA analyses, particularly for the themes: *layout and language style* and *confidence in how to perform the behaviors*. All of the codes which made up the *layout and language style* theme from the human analysis were classified as in agreement with the related codes identified in the MATA. Both methods agreed that Germ Defence users found the website clear to use and easy to understand, but there were a few areas requiring improvement. For example, some users felt that the website did not appear “slick” or sophisticated enough, and that the simple language appeared patronizing to some. Some examples of codes classified as in agreement were: “clear and simple” versus “information was clear, concise and easy to understand”, and too “simplistic/patronizing” versus “did not provide any new information beyond what is already known and is patronizing”.

We also found many instances of agreement between the methods for two of the three codes which made up the theme *confidence in how to perform the behaviors* from the human-only analysis. Both methods agreed that many of the participants felt that the website provided important reminders and reinforcement of the recommended behaviors. For example, for those who were already highly adherent to the behaviors, the website provided assurance that they were doing the right thing and encouragement to continue. For those who experienced difficulty performing the behaviors, the website provided practical guidance and “real-world” examples of how the infection control behaviors could be integrated into users’ daily routines. An example of codes classified as in agreement is “clear practical advice and troubleshooting is helpful” from the human-only analysis versus “helpful information users had not thought of before; the case studies were helpful” from the MATA.

Finally, two of the four codes contained within the *reducing all or nothing thinking* theme agreed with codes generated from the MATA. The majority of the agreement here came from finding that some of the behaviors may be more difficult to integrate, particularly for families with young children. Some participants felt that Germ Defence could appear too proscriptive, and placed emphasis on the need to balance the behaviors according to what was deemed practical and necessary for the family to perform to reduce risk. For example, the ‘some behaviors are very challenging in certain situations’ code from the human-only analysis was classified as in agreement with ‘guidance and questions lack consideration for practicalities within families, especially families with young children’ from the MATA.

#### 3.3.2. Instances of complementarity

The remaining relationships between the findings of the two methods were judged as complementary and there were no instances of dissonance or silence. Only the theme *reducing all or nothing thinking* contained more codes deemed as complementary than in agreement. Both methods found that users placed emphasis on the need to act according to risk level, and that some of the suggested behaviors could be unrealistic in certain households and/or situations. However, the human analysis placed greater emphasis on the potential mental load of integrating the behaviors, and participants’ interpretations of the viral load messages. The viral load messages encouraged some participants by helping them to understand that even small changes (such as implementing some of the behaviors wherever possible and practical, or that they might tailor their behaviors according to risk) can be effective for reducing their risk of catching COVID and/or illness severity. In contrast, believing that they must perform all behaviors perfectly to avoid virus transmission left some participants feeling defeated. The MATA codes did not wholly reflect these interpretations, and so “understanding that small changes matter is motivating” from the human-only analysis was classed as complementary to codes such as: “information on how the virus lives and spreads, along with explanation of the link between amount of viral exposure and severity of illness” from the MATA.

## 4. Discussion

We aimed to explore the potential value of machine learning analysis techniques to analyze large-scale datasets by conducting a comparison between MATA and traditional thematic codebook analysis using a template approach conducted by humans. We triangulated the results of both forms of analysis in order to highlight the similarities and differences between the two methods, and we compared them by the person-hours needed to complete the analyses.

In regard to the primary data, both analyses found that online public health interventions should be clear and concise. For our participants, a slick and professional appearance conveys trustworthiness, and many felt that a website should be uncomplicated and accessible. However, others felt that it seemed overly simplistic and patronizing, indicating a need for striking balance when designing interventions targeted to a wide audience. Rather than simply stating the recommended behaviors, our participants highlighted the importance of practical information and real-life examples which aim to help website users envision *how* the behaviors can be implemented in their own homes. Having the efficacy of the behaviors confirmed by those perceived to be experts empowered participants to act, and reinforced participants’ confidence in their ability to protect themselves and those around them. Finally, our participants indicated that public health interventions should recognize that some of the recommended behaviors can be very challenging in certain situations, and attempting to adhere to all behaviors at all times may not be feasible for many households. Many participants indicated that they would act according to their risk level, and felt that information which appeared overly restrictive and inflexible can leave participants feeling defeated and demotivated. On the other hand, messages which emphasized the concept of viral load helped many participants to understand that making even small changes were worthwhile for reducing viral exposure, and understanding risk reduction as cumulative – rather than absolute – was motivating.

As a result of the triangulation between the two methodologies, we found that the results were very similar, with all codes developed from the MATA classified as in agreement or complementary to the codes developed from the human-only analysis. Where the findings were classified as complementary, this was typically due to slightly differing interpretations or nuance which are likely to be due to the human input to the analyses. For example, the investigator leading the human-only analysis (LT) had analyzed previous Germ Defence data, whereas the MATA team had not. It is therefore likely that LT made interpretations based on knowledge gained from previous analyses of Germ Defence data. This particularly seems to be the case for the codes within the *reducing all or nothing thinking* theme, which were more prominent and developed in the human-only analysis by the Germ Defence team. These concepts were salient to the Germ Defence developers because Germ Defence sought to overcome fatalism about infection transmission. Therefore, some of these differences were likely due to investigator difference, and not methodological difference. That said, the codes from the human-analysis were generally more interpretive than the MATA codes. This is different from the findings from another study which compared human analysis with a different NLP approach. Guetterman et al. (10) found that whilst human-only analysis was of higher quality than NLP-only analysis, a combined approach added further conceptual detail and further conclusions than human-only analysis. We did not find this to be the case in the current study, rather, we found that human-only methods yielded similar results to a human-assisted NLP approach.

One potential consideration is that punctuation is removed for the MATA as only words, rather than phrases or sentences, are used as tokens. Due to the purpose of punctuation being to convey and clarify meaning, emphasis, and tone within text, the human coders may have been able to understand nuances within the responses during the early stages of analysis that could have been missed or misattributed by the AI. However, the role that humans play in understanding and interpreting the output of the MATA means that any potential missed meaning should be minimal. Similarly, the topics produced by STM can sometimes be incoherent, or involve multiple seemingly unrelated themes. This would be a major issue if the goal of this method was to conduct an exhaustive and in-depth qualitative analysis of the corpus. However, since the goal of this analysis, and the use case for MATA in general, was to rapidly extract headline insights, this limitation can be mostly overlooked. Nevertheless, researchers should be mindful of these potential issues when they come to interpret the output of the AI.

Due to these considerations, MATA could potentially be seen as a less interpretive method than human-only analysis that is suitable for more descriptive studies of large datasets. Indeed, the concept of information power recommends larger samples for studies with broader, atheoretical, more exploratory aims (30). In order to complete the human-only analysis of a sample of this size, a codebook was created based on previous Germ Defence research, and six research assistants needed to be trained in qualitative analysis. It would not have been feasible to conduct a purely inductive thematic analysis using a large number of coders due to differences in how individuals would interpret and label the data. Other methods of coding large-scale data, such as crowdsourcing though Amazon Mechanical Turk, have been shown to be successful when coding deductively into pre-determined categories (31–33). However, in the absence of these categories, such as in more inductive approaches or studies with more exploratory aims, there have previously been few options available to researchers other than to perform human analyses on limited sample sizes. Approaches such as MATA could be a valuable tool for enabling large-scale sampling for these types of studies.

In the rapidly evolving landscape of artificial intelligence, the emergence and application of Large Language Models (LLMs) like Chat-GPT in qualitative data analysis needs to be considered as a viable alternative approach. Mellon et al. (34) demonstrated how LLMs can accurately replicate human coding of large-scale data when classifying the most important perceived issues in the United Kingdom, such as health and education. However, whilst LLMs offer scalability and efficiency, it is possible that they could inadvertently introduce biases or miss nuanced interpretations if applied uncritically (35), and researchers must ensure they manually validate the output to verify accuracy and quality. Furthermore, Mellon et al. (34) highlight that it is currently unknown whether LLMs can code the sentiment of open-text data, or whether they are capable of coding the data as well as producing a coding scheme in the way that STM does. It is possible that current and future iterations of Chat-GPT could have these capabilities, but further research is required. The current study demonstrates how a MATA approach can integrate qualitative researcher oversight to ensure sentiment is captured.

Therefore, MATA offers researchers a less resource intensive and time-consuming approach to conducting broader exploratory studies within large, nationally representative samples, whilst still ensuring human oversight in the process to accurately capture meaning and sentiment. It could be used to augment approaches which tend to adopt more descriptive aims such as codebook TA, coding reliability TA, and content analysis. For analyses such as reflexive TA or interpretative phenomenological analysis (IPA) where researchers wish to engage with the data on a richly interpretive level, and the researchers’ knowledge of the subject matter is considered an important analytic lens, we would not currently consider MATA an appropriate approach based on the current findings.

### 4.1. Strengths and limitations

The decision to triangulate human qualitative analysis of Germ Defence data with machine learning analysis was made *post hoc*, and as such, both teams worked and made analytical decisions independently from each other. Whilst this could be seen as a limitation of the current study, we believe that the high level of agreement and complementarity between the two analyses demonstrate the trustworthiness of using machine learning techniques to analyze large-scale datasets. Despite the independence of the two teams, the MATA was still able to generate findings very similar to the human analysis. As discussed above, machine learning techniques may be best suited to more descriptive qualitative analyses, and so it is likely that the results were consistent due to the descriptive aims of the human analysis and the similarity between the results would likely not have been as great if compared with a more interpretive analysis.

The sample of participants in the current study was largely homogenous. The majority of participants were white, midlife or older, and at higher risk of severe illness from COVID-19. We are therefore unable to draw conclusions from the current study as to the utility of MATA and NLP methodology for the analysis of more diverse, nationally representative samples. Further research is needed to assess how NLP techniques handle more diverse datasets.

### 4.2. Conclusion

For studies with more descriptive aims, MATA is a trustworthy and potentially valuable tool to assist researchers analyse large-scale open text data. Previously, qualitative approaches have been limited to small sample sizes by its time-consuming nature. By triangulating the results from a traditional human-only thematic analysis with those from MATA, we have shown that both methods generate comparable findings, whilst MATA has the benefit of being less resource and time intensive. MATA could therefore be used to automate the early familiarization and coding process of more descriptive and less interpretive methods such as codebook analysis or content analysis, especially when the goal is to rapidly extract key topics or concepts from the data for use in a public health emergency. This study contributes to an emerging body of literature into the potential utility of machine learning techniques for use in large-scale qualitative research (5, 8–11).

## Data availability statement

The original contributions presented in the study are publicly available. This data can be found here: https://doi.org/10.6084/m9.figshare.19514305.

## Ethics statement

The studies involving humans were approved by the University of Southampton Psychology Ethics Committee (ID: 56445). The studies were conducted in accordance with the local legislation and institutional requirements. The participants provided their written informed consent to participate in this study.

## Author contributions

LT: Data curation, Formal analysis, Investigation, Methodology, Project administration, Software, Supervision, Validation, Writing – original draft, Writing – review & editing. PB: Data curation, Formal analysis, Investigation, Methodology, Project administration, Software, Supervision, Validation, Writing – original draft, Writing – review & editing. TP: Data curation, Formal analysis, Investigation, Methodology, Project administration, Software, Writing – original draft, Writing – review & editing. RA: Conceptualization, Writing – review & editing. TC: Conceptualization, Writing – review & editing. BA: Conceptualization, Writing – original draft, Writing – review & editing. LY: Conceptualization, Funding acquisition, Writing – review & editing.

## Footnotes

^1^The rationale for exclusion of 3 topics from the analysis was:

- Topic 4 was deemed incoherent
- Topic 11 was described as “Nothing was helpful/Learned nothing new” and hence did not provide a substantial answer to the qualitative question
- Topic 23 included mixed issues that were already represented in other themes.

^2^The rationale for exclusion of 2 topics from the analysis was: Topic 13 was deemed incoherent Topic 15 was described as “Nothing was unhelpful/nothing to dislike” and hence did not provide a substantial answer to the qualitative question.

## Abbreviations

AI: Artificial intelligence
IPA: Interpretative phenomenological analysis
MATA: Machine-assisted topic analysis
NLP: Natural language processing
PBA: Person based approach
STM: Structural topic model
TA: Thematic analysis
