# Supplementary material for: Applying machine-learning to rapidly analyze large qualitative text datasets to inform the COVID-19 pandemic response: comparing human and machine-assisted topic analysis techniques
Source: Front Public Health. 2023 Oct 31;11:1268223. doi: 10.3389/fpubh.2023.1268223 (PMC10644111; doi:10.3389/fpubh.2023.1268223)

**Online supplementary material 2**

Figure 1. Diagnostic values by number of topics for the question A corpus (left) and question B corpus (right)


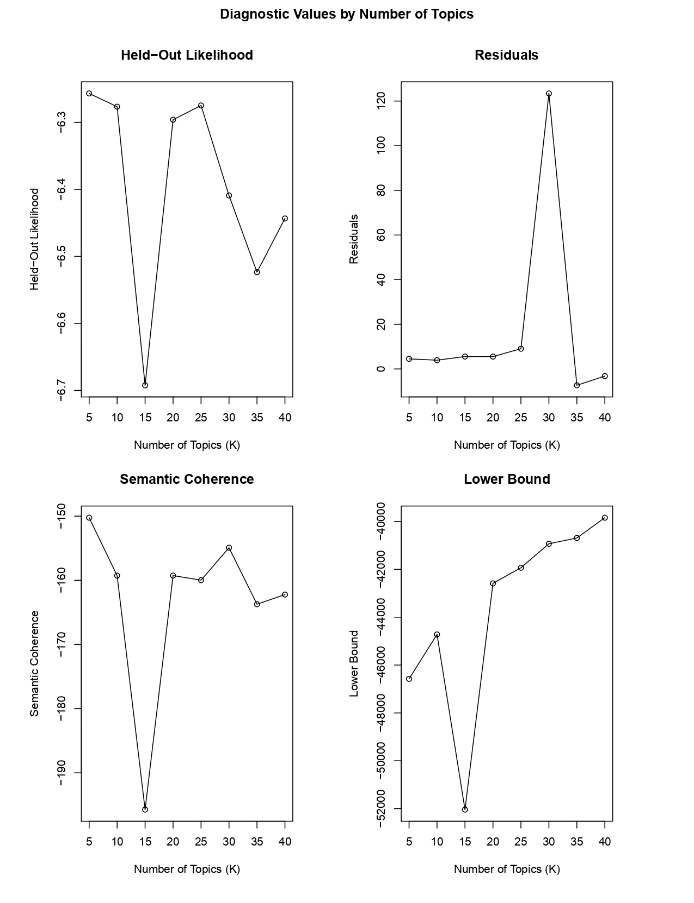

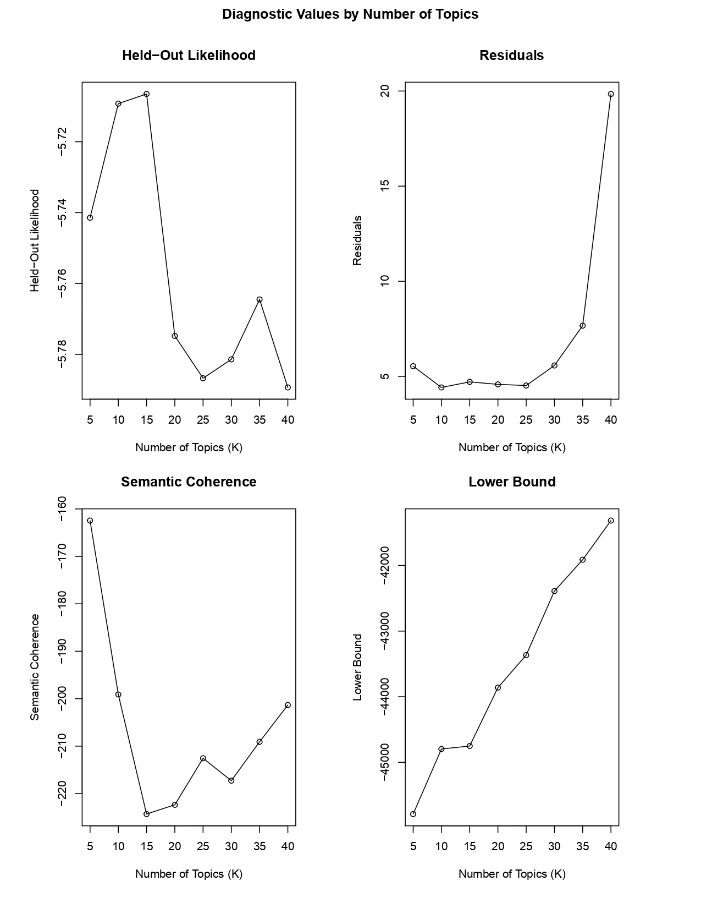

Supplement: Supplementary file 2 [file Table_2.DOCX]
