# Supplementary material for: Applying machine-learning to rapidly analyze large qualitative text datasets to inform the COVID-19 pandemic response: comparing human and machine-assisted topic analysis techniques
Source: Front Public Health. 2023 Oct 31;11:1268223. doi: 10.3389/fpubh.2023.1268223 (PMC10644111; doi:10.3389/fpubh.2023.1268223)

**Online supplementary material 3**

Box 1. Unsupervised machine learning output example on “What was helpful about the information on the Germ Defence website?”


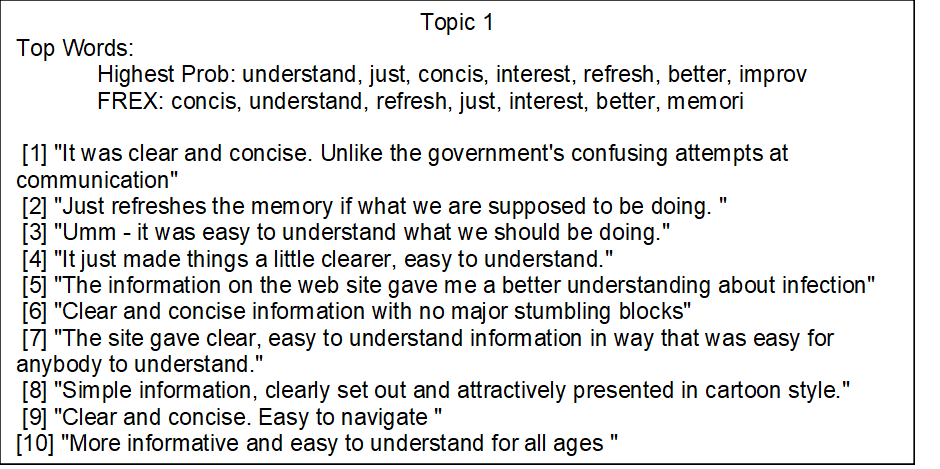


Box 2. Unsupervised machine learning output example on “What did you not find helpful about the information on the Germ Defence website?”


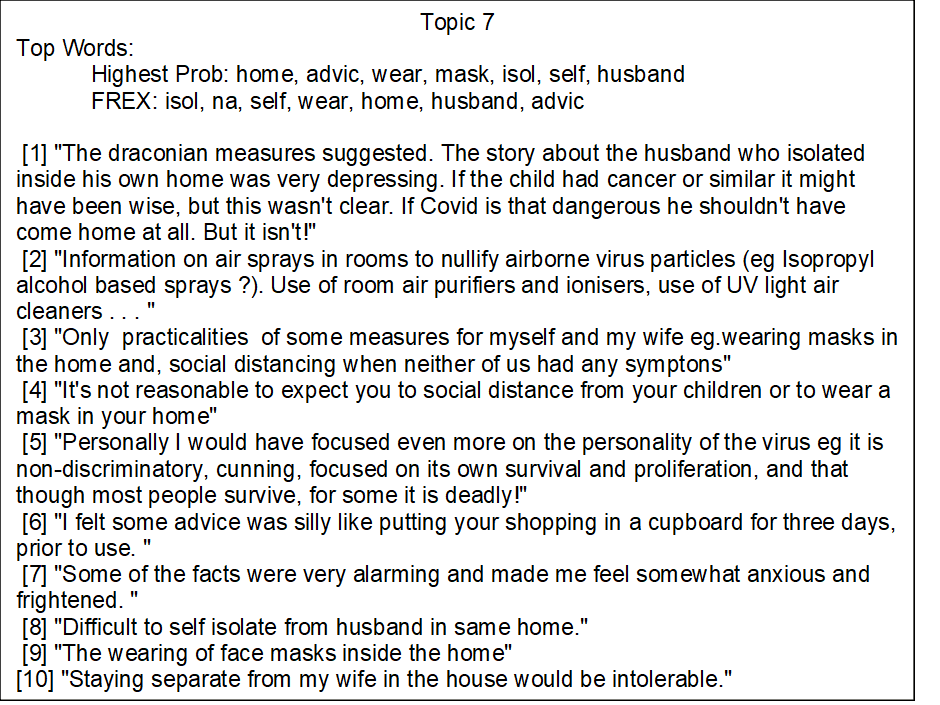

Supplement: Supplementary file 3 [file Table_3.DOCX]
