# Supplementary material for: Applying machine-learning to rapidly analyze large qualitative text datasets to inform the COVID-19 pandemic response: comparing human and machine-assisted topic analysis techniques
Source: Front Public Health. 2023 Oct 31;11:1268223. doi: 10.3389/fpubh.2023.1268223 (PMC10644111; doi:10.3389/fpubh.2023.1268223)
Supplement: Supplementary file 5 [file Table_5.DOCX]

**Online supplementary material 5**

Figure 1. Ranking of topics in terms of prevalence in the corpus for question A, *“What was helpful about the information on the Germ Defence website?”***
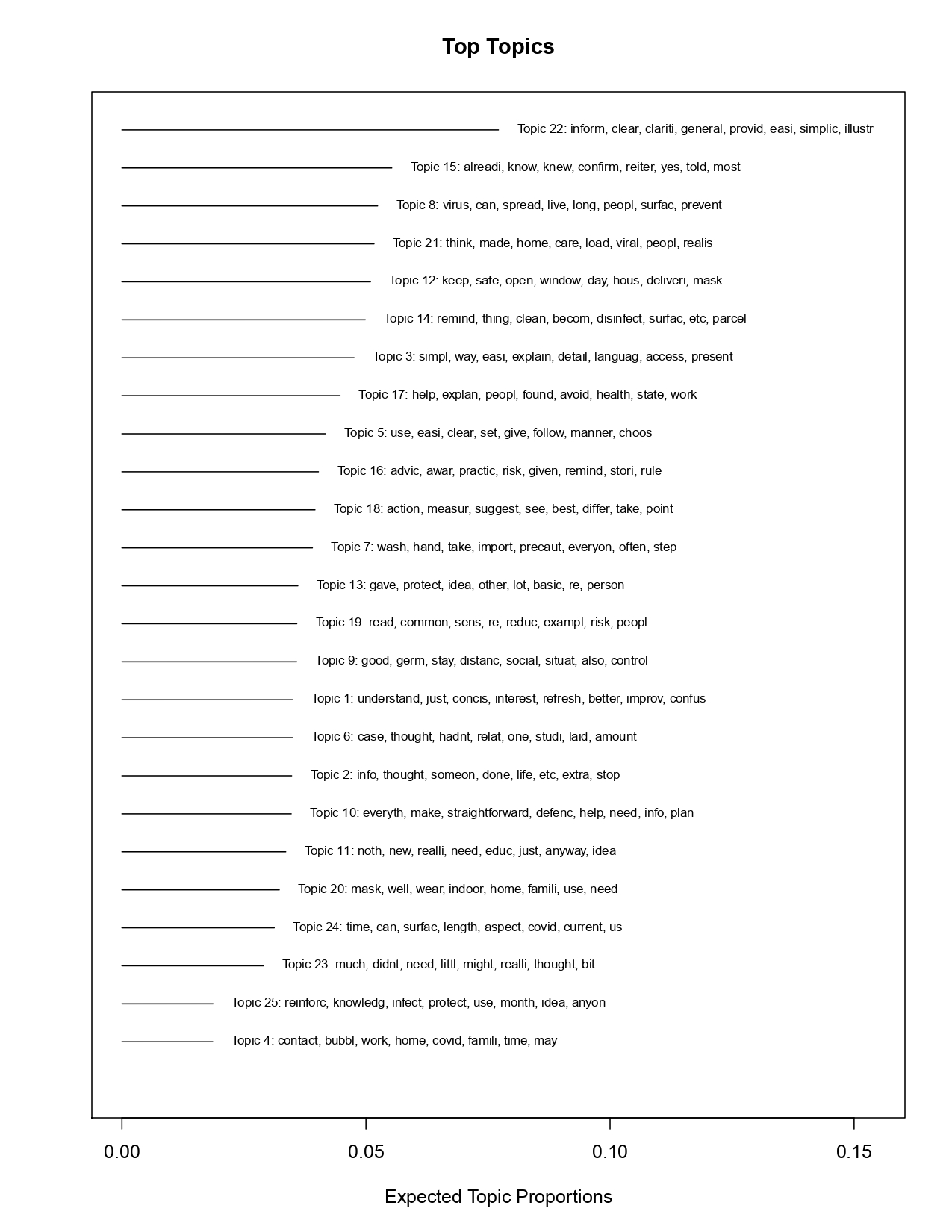
**

Figure 2. Ranking of topics in terms of prevalence in the corpus for question B*, “What did you not find helpful about the information on the Germ Defence website?”*

**
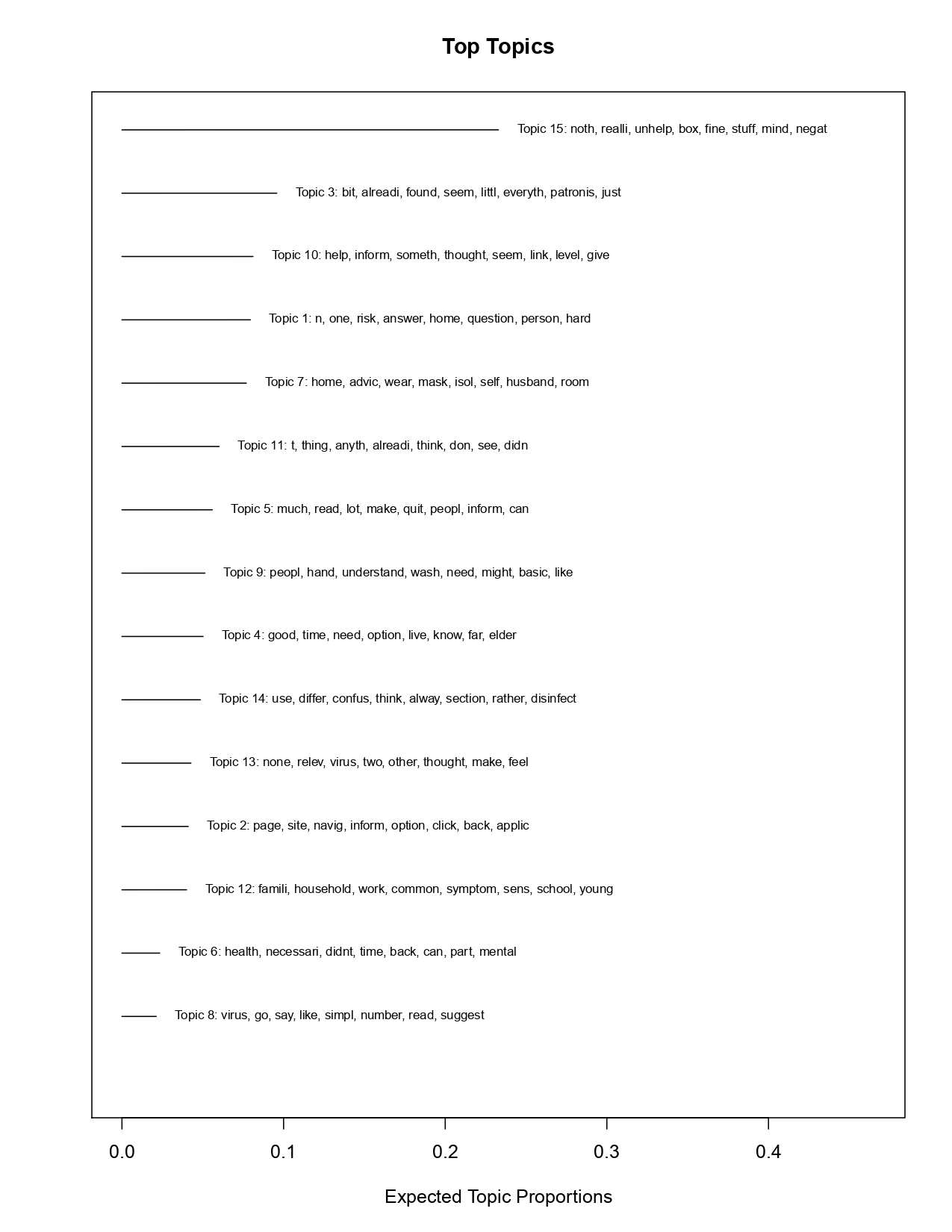
**
